# Ear-Worn Inertial Measurement of Sit-to-Stand and Sit-to-Walk Movements: Algorithm Development and Validation

**DOI:** 10.2196/100936

**Published:** 2026-10-13

**Authors:** Terry Fawden, Amy Edwards, Iwan Vaughan Roberts, Henry Dunne, Thomas Stone, Manohar Bance

**Affiliations:** 1 Department of Clinical Neurosciences University of Cambridge Cambridge, England United Kingdom; 2 Cambridge University Hospitals NHS Foundation Trust Cambridge, England United Kingdom; 3 Cambridge NeuroWorks Frontier Fellow Cambridge, England United Kingdom

**Keywords:** sit-to-stand, sit-to-walk, earables, inertial sensor, mobile health, rehabilitation

## Abstract

**Background:**

Sit-to-stand and sit-to-walk are common functional movements that provide insight into an individual’s functional mobility. Developing comprehensive measures of these movements using ear-worn inertial measurement units (IMUs), which are increasingly incorporated into widely used devices such as hearing aids and headphones, could provide a means of monitoring older adults and individuals with movement disorders with minimal additional burden.

**Objective:**

This paper aimed to introduce comprehensive outcome measures for sit-to-stand and sit-to-walk movements based on data from ear-worn IMUs, and to evaluate their agreement with an optical motion capture reference.

**Methods:**

Data were analyzed from 62 participants, of whom 46 (18 male and 28 female; median age 27, IQR 15 years) did not have a known underlying movement condition and 16 (7 male and 9 female; median age 62, IQR 23 years) did. The participants wore a bespoke 3D-printed headset that contained 6-axis IMUs placed beside their left and right ears. A reflective marker was placed on each participant’s seventh cervical vertebra to provide an optical motion capture reference. Each participant performed 3 sit-to-stand movements and 3 sit-to-walk movements. The reported outcome metrics included overall and subphase durations, acceleration, velocity, power, movement smoothness, and hesitation. Agreement between ear-derived and optical outcomes was evaluated using linear mixed-effects analyses accounting for repeated trials within participants, including systematic and proportional bias, limits of agreement, root mean squared error, and smallest detectable difference.

**Results:**

Temporal outcomes demonstrated the closest agreement between systems. The mean overall movement duration differed by 0.09 (SD 0.12) seconds for sit-to-stand and 0.06 (SD 0.17) seconds for sit-to-walk, with 95% limits of agreement of –0.14 to 0.32 seconds and –0.28 to 0.39 seconds, respectively. Strong linear relationships were observed for peak power, with mixed-effects regression slopes of 0.70 for sit-to-stand and 0.68 for sit-to-walk, although systematic biases of 172 (95% CI 153-190) W and 191 (95% CI 168-215) W indicated that ear-derived and seventh cervical vertebra–derived power were not interchangeable. Peak upward acceleration similarly showed systematic biases of 1.05 (95% CI 0.93-1.18) and 1.30 ms^–2^ (95% CI 1.14-1.45) for sit-to-stand and sit-to-walk, respectively. Hesitation demonstrated comparatively small absolute error (root mean squared error 0.04 and 0.03, respectively), whereas smoothness and other fluency outcomes showed greater disagreement between measurement systems.

**Conclusions:**

The ear-worn IMUs extracted a broad range of sit-to-stand and sit-to-walk outcome measures, although agreement with the optical reference varied between measures. Temporal outcomes demonstrated the closest agreement, whereas kinematic, power, and fluency measures showed varying degrees of systematic and proportional disagreement. These findings support the feasibility of using ear-worn IMUs for comprehensive functional movement assessment and provide a basis for subsequent validation of the most robust outcomes in clinically relevant populations.

## Introduction

Sit-to-stand and sit-to-walk movements are fundamental activities of daily living that are crucial for functional independence [1]. The average person performs around 60 sit-to-stand and sit-to-walk movements per day, despite these being the most demanding functional tasks commonly performed in day-to-day settings. These movements require sufficient muscular strength, coordination, and the ability to balance, with sit-to-walk additionally demanding fluent transition from sit-to-stand to walking gait [2-4]. Sit-to-stand and sit-to-walk performance therefore provides a rich and steady stream of information about an individual’s health, and has already been shown to identify fall risk [5-10], frailty [11-14], osteoarthritis [15], fatigue [16], and rehabilitation progress after stroke [17-19]. Popular clinical tests such as the Five-Times-Sit-to-Stand [20], the Tinetti Balance Scale [21], and the timed up-and-go (TUG) include sit-to-stand and sit-to-walk movements, since they provide a convenient means to assess functional movement capability [22]. However, conventional clinical assessment frequently reduces these complex movements to completion time or relies on subjective clinician observation of movement quality, overlooking much of the useful detail available from deeper quantitative analysis.

Many prior studies involving more detailed sit-to-stand and sit-to-walk movement analysis have been instrumented with force plates [6,14,18,23] and optical motion capture systems [4,17,19,24-41]. While these methods provide detailed kinematic and kinetic data, they are largely restricted to specialist laboratory environments and therefore are poorly suited to repeated measurement in routine clinical practice or daily living scenarios. To alleviate the requirement for specialist equipment, a small body of work has also explored analyzing sit-to-stand using commercially available camera systems [8,15]; however, these methods provide less information while still being restricted by location and assembly time. To address these limitations, other studies have developed methods that rely on wearable inertial measurement units (IMUs)—small, integrated packages containing an accelerometer and gyroscope that report 6-axis acceleration and angular velocity data. These data can be used to derive temporal and kinematic metrics for sit-to-stand and sit-to-walk movements, providing information beyond a simple stopwatch without the restrictive dependence on a specialist motion capture laboratory.

Existing studies have analyzed sit-to-stand and sit-to-walk movements using IMUs placed on the wrist [42], shank [43], and thigh [7,44-48]. The most popular placement studied to date is the trunk, which is desirable as it is anatomically most representative of the body’s center of mass (CoM) trajectory, and both movements are characterized by large trunk flexion-extension [1,5,9-13,16,49-61]. While trunk-mounted sensors are biomechanically convenient, they require a dedicated wearable device to be placed there, imposing an unwanted burden on the user. These approaches also introduce a large opportunity for error due to inaccurate placement by the wearer [62]. As an alternative to existing IMU placement locations, ear-level sensing provides a comparably unobtrusive means to analyze sit-to-stand and sit-to-walk movements. The widespread use of headphones, earbuds, and hearing aids (collectively referred to as “earables”), many of which already incorporate IMUs, provides the opportunity for the measurement to be achieved through existing form factors, eliminating the need for a patient to attach a dedicated sensor in a specific anatomical location. This has already provided the basis for significant investigation into gait analysis using ear- and head-worn sensors, providing comparable and in some cases superior results to other IMU-based methods [63-66].

The head undergoes translational motion during the sit-to-stand and sit-to-walk movements, which early work has found to correlate strongly with trunk movement [67], suggesting that previously developed metrics based on trunk-derived data could be applied to an ear-worn system. However, these metrics have not previously been quantified using an ear-worn system or validated against a reference measurement. Establishing whether ear-derived measures capture valid temporal and kinematic characteristics of these movements is therefore an important step toward integrating sit-to-stand and sit-to-walk assessment into ear-worn devices.

This study aimed to quantitatively validate the agreement between sit-to-stand and sit-to-walk metrics derived from ear-worn IMUs and an optical motion capture reference across a range of movement characteristics. These included the time to complete the total movement and each of its subphases, kinematic measures such as power, peak acceleration, peak velocity, and exploratory measures of movement fluency. In tandem, a sensitivity analysis was performed using a subset of participants with optical markers placed on top of the ear-worn IMUs to establish the extent to which disagreement with the trunk-mounted optical reference could be attributed to differences in anatomical measurement location.

## Methods

### Ethical Considerations

This study was conducted in accordance with the Declaration of Helsinki and approved by the Ethics Committee of Cambridge University Hospitals NHS Foundation Trust (23/EM/0145 and September 2023) for studies involving humans.

### Participants

Participants were recruited via physical and electronic advertisements around Cambridge Biomedical Campus and through word-of-mouth. Exclusion criteria were being younger than 18 years of age and being unable to safely walk 10 m unaided. A total of 65 participants were recruited, of whom none were excluded due to not meeting the criteria, although 3 were subsequently excluded due to poor optical marker data quality. Of the 62 participants analyzed, 16 had an underlying condition that affected their movement. This included 8 participants with Parkinson disease, 3 participants who had recent vestibular schwannoma surgery, 1 participant with benign paroxysmal positional vertigo, 1 participant with Ménière disease, and 3 participants with osteoarthritis. Additional participant details are shown in Table 1.

In total, 395 movement trials were recorded. Following the exclusion of 31 sit-to-stand trials and 28 sit-to-walk trials due to poor optical marker data quality, sit-to-stand data were available for 163 trials (59 participants) and sit-to-walk data were available for 173 trials (61 participants). The disparity between the number of participants analyzed for sit-to-stand and sit-to-walk data was due to certain participants only providing clean marker data for either sit-to-walk or sit-to-stand. In parallel with the main analyses, a subset of 13 participants had optical markers placed on top of the ear-worn IMUs (though one of these participants only recorded clean sit-to-walk data), and data from these trials were used to conduct an anatomical-location sensitivity analysis.

**Table 1:** Summary of participant demographic information.

Characteristic	Typically functioning	Known movement disorder
Sex (male/female), n	18/28	7/9
Age (years), median (IQR)	27 (24-39)	62 (52-75)
Weight (kg), median (IQR)	62 (56-73)	82 (66-89)
Height (m), median (IQR)	171 (165-180)	170 (164-178)

### Instrumentation

All data were collected on an 8 m long walkway in the Cambridge Clinical Movement Laboratory, using a standard chair with armrests that was placed in the center of the walkway. Each participant wore a bespoke headset (known as the “earable”) that housed two 6-axis LSM6DS0x IMUs placed beside the left and right ears. The headset weighed 25 g, including the band (dimensions 3 mm × 7 mm × 250 mm) and IMU enclosures (5 mm × 19 mm × 27 mm), and was 3D printed using Formlabs Tough 1500 resin (flexural strength 39.0 MPa). The IMUs were wired to an ESP32 Thing Plus board (SparkFun Electronics), which collected the accelerometer and gyroscope data from the ear-worn IMUs and an identical IMU placed at the chest at 100 Hz via I2C protocol. The Thing Plus board and chest IMU were held comfortably and securely in place by an elasticated chest strap. The Thing Plus board was powered by a 180 mAh lithium polymer battery and sent data to a laptop via Wi-Fi to be processed offline.

Along with the IMUs, each participant had an optical marker placed on the prominent spinous process of their seventh cervical vertebra (C7), palpated at the base of the neck, while a subset of 13 participants had additional markers placed on each IMU. The C7 placement was chosen as previous work by Chandler et al [17] had found that the C7 marker remained reliably in view throughout each sit-to-stand and sit-to-walk trial, while the C7 data demonstrated a correlation of 0.95 with the CoM data in a cohort of patients who had stroke. Tracking the CoM directly requires several markers to remain visible throughout the movement, which was difficult in the present study as some markers were occluded when the participants were sitting or in a flexed position. The data from this C7 marker were therefore selected to act as an optical reference measurement to compare with the earables, and a sensitivity analysis was performed using data from those participants with optical markers placed directly on the headset to separate IMU-vs-optical disagreement from anatomical-location disagreement.

The optical markers were captured by the optical motion capture system (Vicon) with 12 infrared cameras, and 2 RGB (red, green, and blue) cameras placed in front of and to the side of the walkway, respectively. The IMUs and camera system were triggered at the same time, and any small differences in start time were corrected by maximizing the cross-correlation between them. The placement of the IMUs and optical markers is demonstrated in Figure 1.

**Figure 1:**
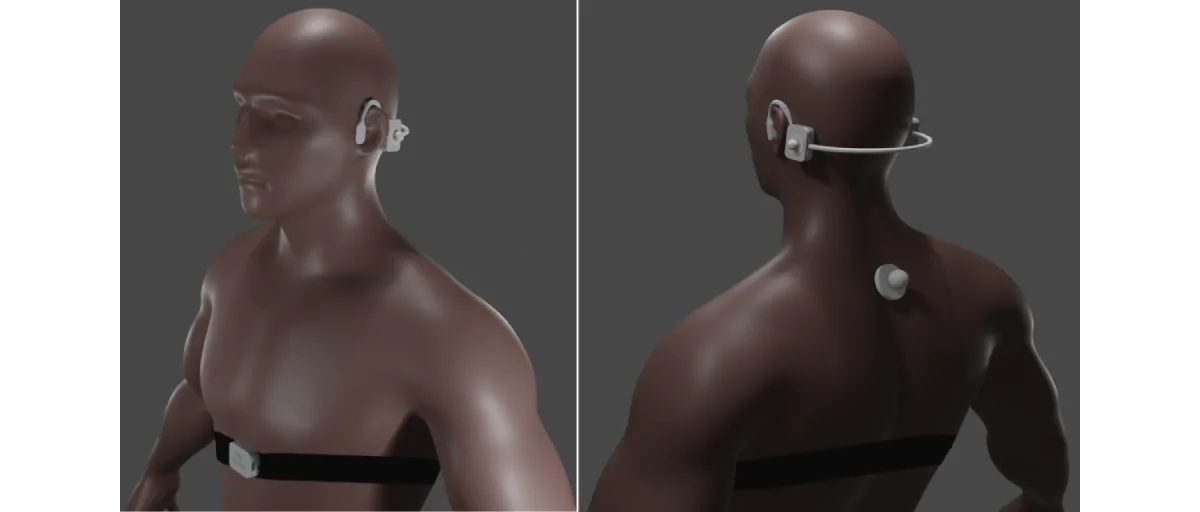
A model demonstrating the placement of the ear- and chest-worn IMUs and the placement of the optical markers on the C7 and headset. C7: seventh cervical vertebra; IMU: inertial measurement unit.

### Experimental Protocol

Each participant completed 3 sit-to-stand and sit-to-walk trials. They began seated on the chair with both feet on the floor and began the movement when verbally instructed. For the sit-to-stand trials, the participant stood up from the chair and remained standing at the end of the trial. From this position, a separate recording was then made for the stand-to-sit movement, which is not discussed here. This process was repeated 3 times.

The sit-to-walk trials were collected as part of a TUG trial, during which the participants were instructed to leave the chair, walk up to and around a cone placed 3 m away, then return to the chair and sit down. Participants were instructed to complete the trial as quickly and as safely as possible. The sit-to-walk trial was defined as the period from initiation of rising until the first foot contact event, as defined by Chandler et al [17]. For both sit-to-stand and sit-to-walk, the participants were instructed not to use their arms to propel themselves from the chair but could otherwise choose their own strategy.

### Signal Processing Pipeline

#### IMU Preprocessing

The IMU data were collected at 100 Hz, then low-pass filtered to 6 Hz using a second-order Butterworth filter. This cutoff frequency was used by Chandler et al [17] in their study of sit-to-stand and sit-to-walk movements, and was found to produce good signal quality for both the optical and IMU data without overly attenuating their key features.

#### IMU Stationary Calibration

The IMU coordinate frame was transformed to align with an east-north-up frame. This corresponded with the optical system frame while enabling direct comparison with previous work, which defined the “rising” movement to be positive. To do this, a stationary time preceding each trial was identified as a time period for which the absolute magnitude of the gyroscope was below 0.05°s^–1^, in a similar approach to Tateoka et al [49]. Then, the roll axis of coordinate frame of the IMU was aligned by transforming the accelerometer readings to match the gravitational vector (ie, to read 0 ms^–2^), which matched the plane of the sit-to-stand and sit-to-walk movements.

After an initial orientation of the sensor was estimated using the accelerometer data, the sensor fusion algorithm introduced by Mahony et al [68] was applied to determine the orientation of the sensor throughout the movement. The algorithm and parameters (*k_p_*=0.01 and *k_i_*=0.001) were selected following a systematic comparison with other algorithms. The gravitational component was then subtracted from the acceleration signal to produce the linear acceleration of the IMU.

Applying the assumption that forward velocity was 0 during the stationary period, the pitch estimate was tuned using the data acquired during the stationary period to minimize the mean forward linear acceleration. This mitigated drift artifacts appearing in the velocity signal calculated in the next stage of the pipeline.

#### IMU Velocity Calculation

The forward and upward velocities were calculated via numerical integration of the linear acceleration signals. This was achieved using the cumulative trapezoid (*cumtrapz()*) function from the SciPy library. For the sit-to-stand trials, an additional step was taken to subtract any drift in velocity over the course of the movement, as it was known that the participant began and ended with approximately 0 velocity [5]. Drift was estimated to act as a straight line from 0 at movement onset to the residual velocity at the end of the signal. This estimated drift was subtracted sample-by-sample from the integrated velocity signal, thereby constraining the corrected velocity to 0 at both the beginning and end of the analyzed interval. The process is demonstrated for an example trial in Figure 2.

**Figure 2:**
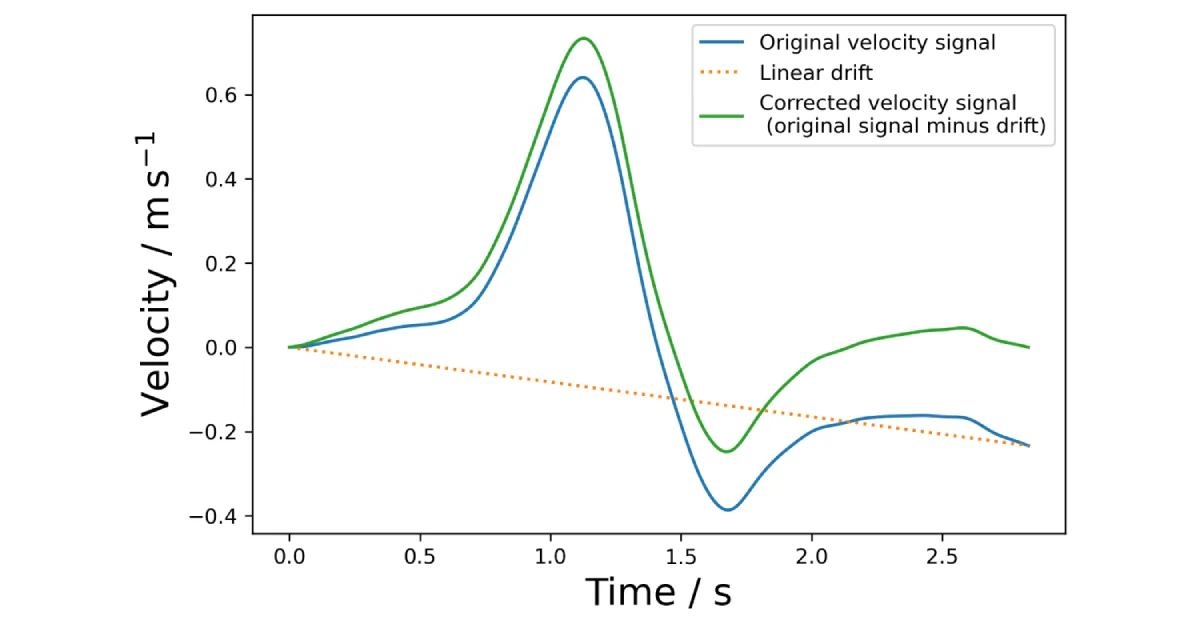
Linear drift correction applied to an upward velocity signal collected during an example sit-to-stand trial. Drift was modeled as acting linearly throughout the movement and was removed by applying the constraint that each movement began and ended with 0 velocity.

### Optical Preprocessing

The optical marker trajectories were collected at 100 Hz from the Vicon optical motion capture system, then filtered to 6 Hz using a second-order Butterworth filter, matching the IMU data. Each marker trajectory was differentiated and divided by the sample time to produce a velocity signal. This was then repeated to produce linear acceleration.

### Formatting Signals for Comparison

The data from the left and right ear-worn IMUs were combined by taking the mean—this was considered to be valid as the movement primarily took place in the sagittal plane with limited axial rotation. In the handful of cases where the participants looked at the person running the data collection (to the side) before straightening their head and performing the movement, the averaged signal still effectively represented the trajectory of the center of the head. The data from the optical markers placed on the ear were also combined by taking the mean, allowing for direct comparison between the IMU and optical data during the sensitivity analysis.

When analyzing the sit-to-walk trials, the optical data were cropped to end at the time when the participant’s first step contact was observed using the RGB cameras. Two copies of the IMU data were kept—one uncropped, which was used to identify the estimated end point of the movement (ie, first step contact) based on the IMU data alone, and another that was cropped to the time of first step contact observed by the camera (ie, that used by the optical data) to enable the metrics derived from the IMU and optical data to be compared directly without the additional errors introduced by differences in the ending. The calculation of these metrics is described in the next section.

### Definition of Movement Phases

#### Overview

While the definition of phases varies greatly in existing work, we chose to use the definitions defined by Kerr et al [24] and extended to sit-to-stand by Chandler et al [17], as they used features that were simple to extract from IMU data alone. The phases are illustrated in Figure 3 and were defined as follows. Regarding the flexion-momentum, after initiating the movement, the participant flexes their trunk forward to position the CoM and generate the initial (predominantly forward) momentum to rise. Regarding the extension, the trunk and lower limb joints extend, propelling the CoM upward. Regarding the unloading, after reaching peak upward velocity, the body continues to extend upward. For sit-to-stand, this constitutes CoM deceleration until the participant reaches a fully upright position. For sit-to-walk, this phase constitutes the initiation of gait, ending when the foot leaves the floor. (4) Regarding the stabilization or stance, for sit-to-stand, the participant stands upright until they have reached stable balance. For sit-to-walk, the participant fully transitions to gait, with the phase ending at initial contact of the swinging foot with the floor.

**Figure 3:**
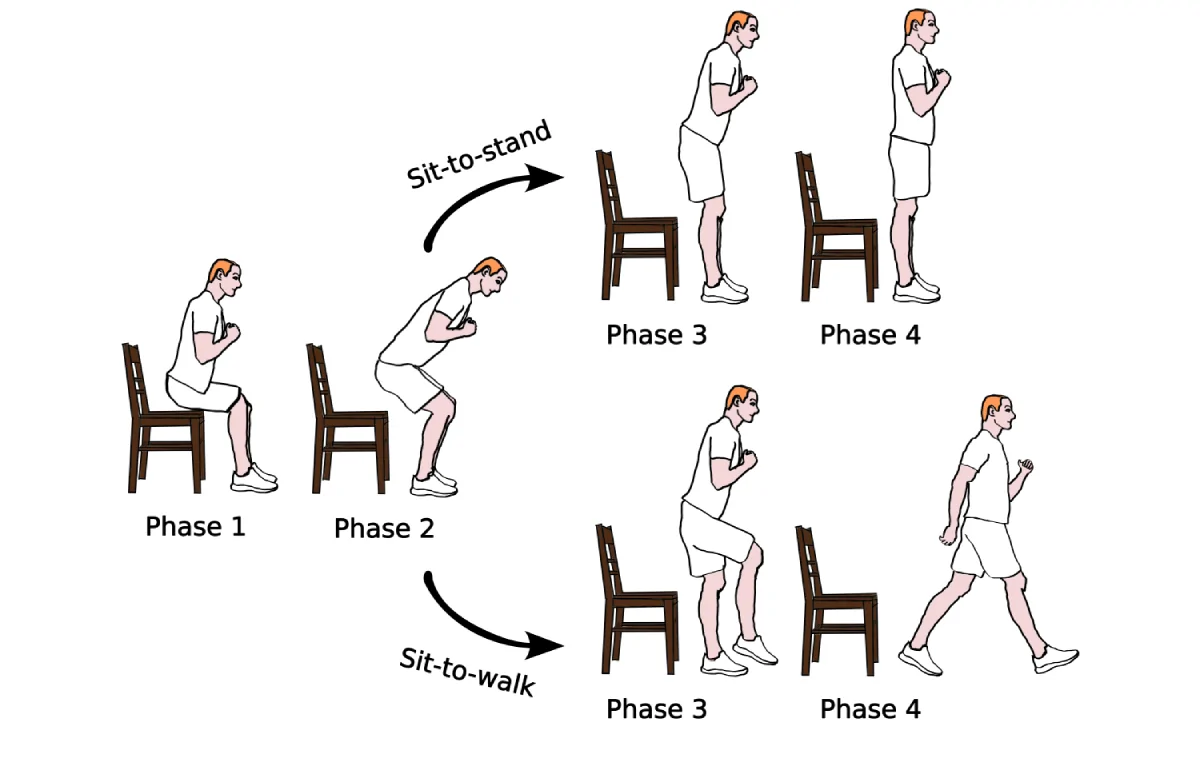
The phases of the sit-to-stand and sit-to-walk movements. Phases 1 and 2 are common to both, though phases 3 and 4 differ as sit-to-walk includes initiation of gait.

#### Identification of Movement Phases

##### Start

There is not currently a universal definition that determines when sit-to-stand and sit-to-walk movements have started, and proposed definitions vary depending on which sensing modality was used. As IMUs produce raw acceleration data, and acceleration can be easily derived from the optical marker trajectory, it was decided in this study to define the start of both movements via an acceleration threshold. The movement commences with trunk flexion; hence, forward acceleration was used, with the threshold determined empirically to be 0.2 ms^–2^, which matched the approximate start of the movement determined by manual inspection of video (recorded in synchronization with the optical motion capture system). To avoid false positives caused by participants nodding their head or shuffling in their seat, the condition was set that the following 20 samples (0.2 s) after crossing the threshold were also above 0.2 ms^–2^.

##### Sit-to-Stand End

Similarly, the precise end point for sit-to-stand and sit-to-walk movements is not universally defined. This study defined the ending for the sit-to-stand movement as the first time after movement initiation that the upward acceleration had performed a positive 0 crossing (indicating that the participant was fully upright) and the rolling SD of the absolute acceleration across 10 samples was less than 0.05 ms^–2^ when filtered to 2 Hz. The latter condition was selected as swaying predominantly takes place below 1 Hz, so this mitigated the effects of any unrelated head movements [69].

##### Sit-to-Walk End

For sit-to-walk, the end was defined as the initial contact of the swing foot, constituting the first step of gait. Chandler et al [17] used the first step when calculating hesitation, a measure that requires time normalization; hence the authors chose the first step to allow direct comparison between our work and theirs. Additionally, detecting initial contact events is more reliable when using accelerometry compared to alternatives such as detecting the first stance toe-off [24,70]. The timing of the initial contact was defined as the first upward acceleration peak following the positive 0 crossing that was greater than 1 ms^–2^—this threshold was determined to differentiate between simple head accelerations during the unloading phase and the initial contact. This was compared to the timing of the first step identified manually using the camera data.

##### Phases 1-4

The key events used to segment the phases of the sit-to-stand and sit-to-walk movements are shown in Table 2. The movement phases used in this study were adapted from the definition by Kerr et al [24] to be more suitable for use with the IMU signals, namely by replacing peak upward force with peak upward acceleration to define the start of phase 2 (extension) and another local upward acceleration peak to define the end of sit-to-walk. These events are labeled in Figure 4 [17,34], which shows data obtained from the earables system during sit-to-stand and sit-to-walk sample trials.

As the authors were unable to determine a reliable acceleration- or velocity-based method for determining the moment at which the foot left the floor during the sit-to-walk trials, the unloading and stance phases were not segmented. This remains a limitation of ear- and trunk-worn IMU-based methods, though future work may explore analysis of the medio-lateral acceleration or gyroscope roll signals, as medio-lateral force plate readings have been used to identify when the foot has left the floor [24]. Therefore, this work focused on the analysis of the flexion-momentum (phase 1) and extension (phase 2) phases for both sit-to-stand and sit-to-walk, and the unloading (phase 3) and stabilization (phase 4) phases for the sit-to-stand task only.

**Table 2:** The start and end definitions for the movement phases. “Initiation” and “end” correspond to the start and end definitions for the sit-to-stand movement defined in the section Overview under Definition of Movement Phases. Phases 3 and 4 were only defined for the sit-to-stand movement.

	Phase 1: flexion-momentum	Phase 2: extension	Phase 3: unloading (STS^a^)	Phase 4: Stabilization (STS)
Start	Initiation	Peak upward acceleration	Peak upward velocity	See phase 3 end in this table
End	Peak upward acceleration	Peak upward velocity	First positive 0 crossing in the upward acceleration signal after phase 2	End

^a^STS: sit-to-stand.

**Figure 4:**
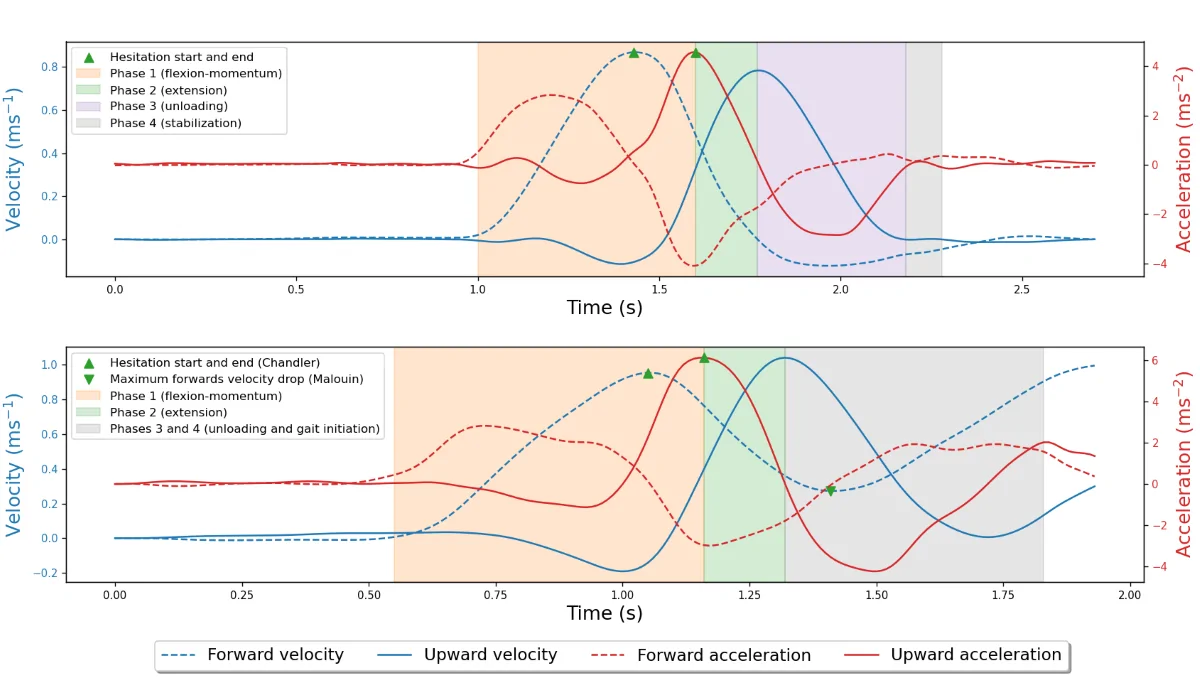
Example signals obtained from the earables during a single sit-to-stand (top) and sit-to-walk (bottom) movement. The shaded areas correspond to each movement phase, while the green triangles correspond to key points relevant to hesitation calculation. The hesitation definition by Chandler et al [17] was calculated as the percentage time between the 2 upward green triangles (peak forward velocity and peak upward acceleration). The definition by Malouin et al [34] for sit-to-walk was calculated as the percentage drop in forward velocity between the first green upward triangle (peak forward velocity) and the green downward triangle (the “trough” in forward velocity).

#### Outcome Metrics

##### Movement and Phase Durations

The durations of the total movement and movement phases were calculated from the processed signals using the events detailed in Table 2 and Figure 4. Specifically, total duration was defined as the time interval between movement start and movement end, while the durations of phases 1-4 corresponded to the time interval between the start and end events detailed for each phase.

##### Power, Acceleration, and Velocity

For each trial, power was estimated by the calculation:



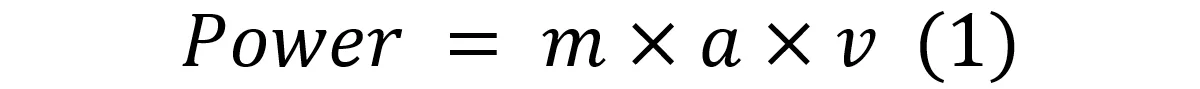



where *m* was the mass of the participant, *a* was the upward acceleration, and *v* was the upward velocity calculated as described in the Signal Preprocessing Pipeline section. Note that power refers to whole-body power as opposed to an individual body segment.

A regression analysis was performed on the peak and mean power reported by the earables against those calculated from the C7 marker trajectory. The strength of the linear relationship was determined using a linear mixed-effects regression with the C7-derived values as the dependent variable, the ear-derived values as the fixed effect, and the participant as a random intercept to handle repeated trials. The fixed-effect slope was used to evaluate the adjustment factor required to correct the readings from the ear to those of the optical reference. This process was repeated for peak and mean upward velocities and accelerations.

##### Fluency

Movement quality is an important component when assessing how a movement has been performed, and several metrics have been developed to characterize this during sit-to-stand and sit-to-walk movements. We performed an exploratory analysis for 2 movement fluency metrics: hesitation and smoothness.

Hesitation seeks to describe the maintenance of forward momentum throughout the sit-to-walk movement, and this idea was extended to the sit-to-stand movement by Chandler et al [17] to enable comparison across the 2 movements. Meanwhile, smoothness describes the continuity of a movement considered independently of its amplitude and duration [71]. This is perhaps best encapsulated by its antonym—jerkiness—and, in fact, many smoothness metrics such as those used by Kerr et al [32] and Balasubramanian et al [71] are functions of the movement signal’s jerk.

##### Hesitation

Hesitation scores were calculated using data from both the earables and the C7 marker, following the modified definition by Chandler et al [17] to allow direct comparison between the sit-to-stand and sit-to-walk movements. This was done by identifying the peak upward acceleration and peak forward velocity from the signals using SciPy’s *argrelextrema()* function, then calculating the time between them as a percentage of the overall movement duration. The timing of peak upward acceleration and peak forward velocity are shown as the green upward triangles on Figure 4.

Additionally, it was decided to implement the hesitation definition by Malouin et al [34] (the percentage drop in forward velocity) for the sit-to-walk movement, as this captured the concept of forward momentum conservation that underpins the transition from rising to gait. In Figure 4, peak forward velocity is indicated as a green upward triangle (as this was common with the definition by Chandler et al [17]) and the drop in forward velocity is indicated by a green downward triangle.

##### Smoothness

Smoothness was defined using 2 metrics: first, by counting the number of inflection points in the forward jerk signal, as used by Chandler et al [17] and Kerr et al [32], and second, using the log dimensionless jerk (LDLJ) metric. First introduced by Balasubramanian et al [71], LDLJ avoids large jumps in smoothness for movements that include few submovements, as is the case for sit-to-stand and sit-to-walk in individuals with good movement function. While standard LDLJ takes the velocity signal as input, we used the IMU-specific version (LDLJ-A) introduced by Melendez-Calderon et al [72], which takes acceleration as input, thereby avoiding issues caused by integrating the accelerometer signal. The calculation of LDLJ-A is shown in equation 2.



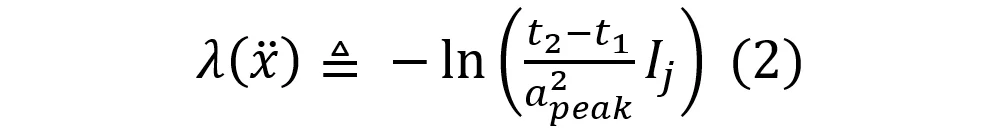





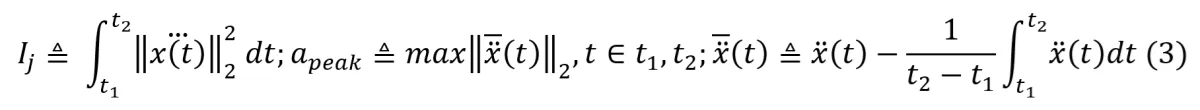



where is the raw acceleration signal from the accelerometer, is the mean-subtracted acceleration, is the maximum absolute acceleration, and is the jerk integral term.

#### Statistical Analysis

##### Scalar Agreement and Linear Mixed-Effects Models Analysis

Comparison statistics were calculated for the difference between the ear-worn IMUs and the C7 marker, incorporating all processed trials. To account for the fact that multiple trials were collected per participant, a linear mixed-effects model was implemented with the participant set as the random intercept. This was applied to all reported metrics and was calculated using the following equation:



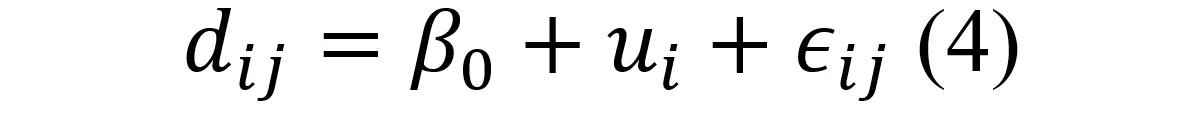



where *β*_0_ is the population-level systematic bias, 
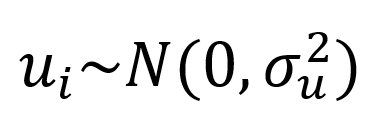
 is the participant-specific random intercept, and 
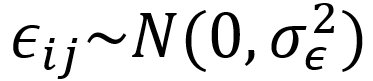
 is the residual intraparticipant error.

For each of the outcome metrics, the following comparison statistics were calculated between the ear-worn IMUs and the C7 marker.

##### Bias

The bias was calculated as the observed fixed intercept, 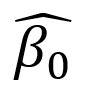 of the linear mixed-effects model.

##### 95% Limits of Agreement

The 95% limits of agreement were calculated from the linear mixed-effects model using the following equation:



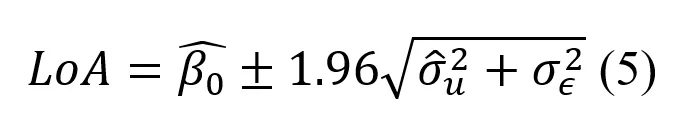



where 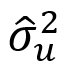 represents the observed interparticipant variance and 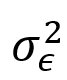 represents the observed intraparticipant variance.

##### Root Mean Squared Error

The root mean squared error was calculated using participant weighting to ensure that a given participant contributing more trials was not given more weight. The equation for this was:



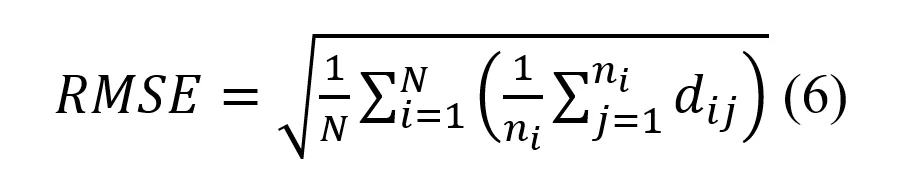



where *N* is the number of participants, *n_i_* is the number of trials contributed by a particular participant, and *d_ij_* is the paired difference calculated between the IMU and optical marker for a given trial.

##### Proportional Bias

Proportional bias between the IMU and optical measurements was tested by fitting a second random intercept mixed-effects model with the following equation:

*d_ij_* = *β*_0_ + *β*_1_*m_ij_* + *u_i_* + *∈_ij_*

where 
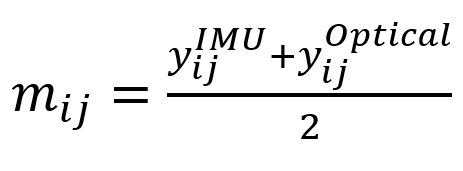
 was the mean of each paired IMU and optical measurement. The sign and magnitude of the observed proportional bias coefficient, 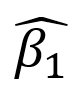 models the presence of any proportional bias between the IMU and optical measurements. A hypothesis test was conducted with the null hypothesis 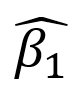
=0 to determine whether significant proportional bias was observed.

##### Smallest Detectable Difference

The smallest detectable difference was estimated from the intraparticipant variance as 
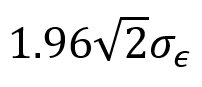
 to describe the smallest difference that can be statistically detected from the data.

#### Supporting and Sensitivity Analyses

##### Ear IMU-C7 Marker Anatomical-Location Analysis

Although the present work focuses on analyzing the sit-to-stand and sit-to-walk movements using ear-worn IMUs, the main optical reference used in this study was placed on the C7. This introduced anatomical-location disagreement alongside IMU-vs-optical disagreement. To characterize these effects, agreement metrics were calculated between the IMU and optical time-series signals recorded for each movement. Bias, root mean squared error (RMSE), and Pearson product-moment correlation coefficients were calculated between the IMU and C7 marker data for velocity and acceleration in the forward and upward directions. For the subset of 13 participants with optical markers placed directly on the headset, the agreement between the ear-worn IMUs and the headset markers was calculated in the same way. This enabled a threefold comparison: ear-worn markers vs C7 marker, ear-worn IMUs vs ear-worn markers, and ear-worn IMUs vs C7 marker, which allowed the effects of anatomical location and IMU-vs-optical disagreements to be isolated.

##### Failed Event Detections

While analyzing the phase durations, it became apparent that the first step of the sit-to-walk movement was misdetected by the IMUs for some trials. This is an important event as it defines the end of the movement, and indeed sit-to-walk’s clinical relevance comes from capturing the transition from rising to gait initiation. To quantify the effect of these misidentified steps on the other outcome metrics, the metrics were calculated both including and excluding the failed detections, and the differences were compared.

## Results

### Phase Durations

The outcome metrics related to overall duration and durations of phases calculated using the C7 data and the earables data are shown in Table 3. The mean overall durations were comparable between both systems, with a mean bias of 0.09 (SD 0.12) s for sit-to-stand and 0.06 (SD 0.17) s for sit-to-walk (Figure 5). Mean biases were low across the phases, though as phase 4 was much shorter than the other, its trial-level error was relatively larger. Figure 6 demonstrates that most trials lay within the limits of agreement for both sit-to-stand and sit-to-walk overall duration.

**Table 3:** Summary of outcome metrics calculated from the overall and phase durations for sit-to-stand and sit-to-walk.

Activity and duration (s)			Earable, mean (SD)		C7^a^, mean (SD)		Bias (95% CI)		Lower 95% LoA^b^ (95% CI)		Upper 95% LoA (95% CI)		RMSE^c^		Proportional bias				SDD^d^
															β (95% CI)		Holm *P* value		
Sit-to-stand																			
	Overall	1.62 (0.26)		1.53 (0.25)		0.09 (0.07 to 0.11)		–0.14 (–0.17 to –0.10)		0.32 (0.27 to 0.37)		0.15		0.06 (–0.02 to 0.13)		.81		0.28	
	Phase 1	0.72 (0.12)		0.69 (0.12)		0.03 (0.02 to 0.04)		–0.08 (–0.10 to –0.07)		0.15 (0.12 to 0.17)		0.07		–0.05 (–0.13 to 0.03)		.83		0.15	
	Phase 2	0.25 (0.08)		0.23 (0.07)		0.02 (0.01 to 0.02)		–0.07 (–0.09 to –0.06)		0.10 (0.09 to 0.12)		0.05		0.01 (–0.08 to 0.10)		.99		0.12	
	Phase 3	0.58 (0.13)		0.54 (0.14)		0.04 (0.03 to 0.06)		–0.17 (–0.23 to –0.11)		0.25 (0.19 to 0.31)		0.11		0.03 (–0.09 to 0.14)		.99		0.29	
	Phase 4	0.07 (0.07)		0.07 (0.06)		0.00 (–0.01 to 0.02)		–0.16 (–0.18 to –0.13)		0.17 (0.14 to 0.19)		0.08		0.22 (0.04 to 0.39)		.09		0.22	
Sit-to-walk																			
	Overall	1.44 (0.32)		1.39 (0.30)		0.06 (0.02 to 0.10)		–0.28 (–0.32 to –0.21)		0.39 (0.27 to 0.50)		0.18		0.13 (0.04 to 0.21)		.02		0.29	
	Phase 1	0.68 (0.13)		0.65 (0.12)		0.02 (0.02 to 0.04)		–0.08 (–0.12 to –0.05)		0.13 (0.09 to 0.18)		0.06		0.07 (0.00 to 0.14)		.13		0.13	
	Phase 2	0.20 (0.07)		0.19 (0.07)		0.01 (0.00 to 0.02)		–0.10 (–0.15 to –0.04)		0.12 (0.06 to 0.17)		0.06		0.19 (0.06 to 0.32)		.02		0.13	
	Phase 3^e^	0.57 (0.21)		0.55 (0.20)		0.02 (–0.01 to 0.06)		–0.31 (–0.35 to –0.24)		0.35 (0.23 to 0.45)		0.17		0.24 (0.11 to 0.36)		.002		0.31	

^a^C7: seventh cervical vertebra.

^b^LoA: limits of agreement.

^c^RMSE: root mean squared error.

^d^SDD: smallest detectable difference.

^e^The data reported for sit-to-walk phase 3 includes phases 3 and 4 combined.

**Figure 5:**
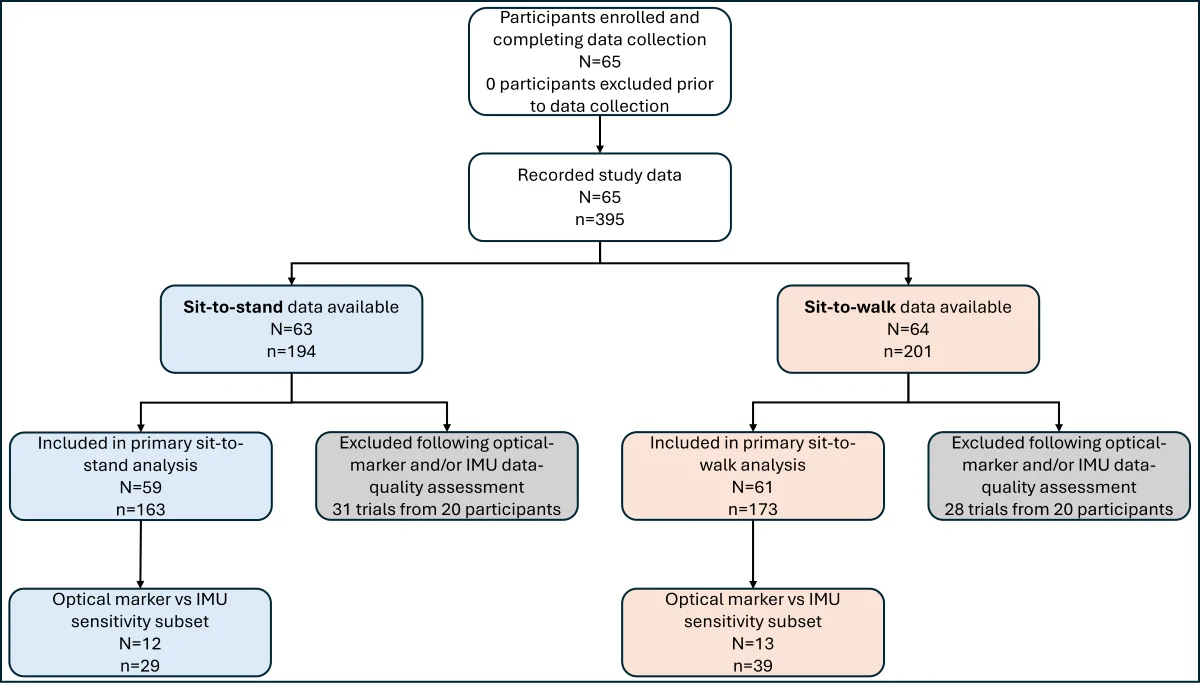
Participant and data availability flow throughout this study and analysis. N denotes the number of participants and n denotes the number of trials. IMU: inertial measurement unit.

**Figure 6:**
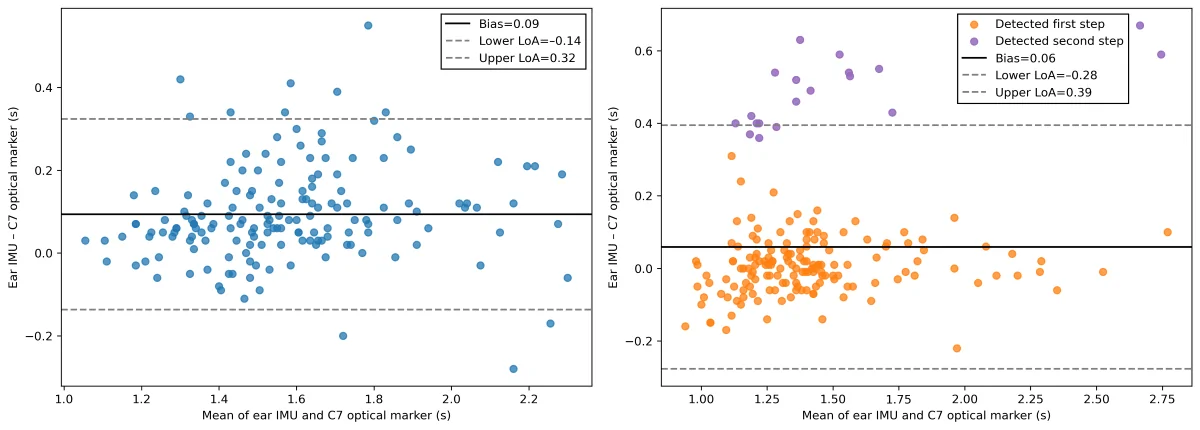
Comparison of total trial duration calculated by the earables and C7 marker data for sit-to-stand and sit-to-walk. The data points marked in purple for sit-to-walk indicate cases where the algorithm missed the first step. C7: seventh cervical vertebra; IMU: inertial measurement unit; LoA: limits of agreement.

### Influence of Failed Event Detection

Inspecting Figure 6, there is a cluster of overestimates in the region of 0.5 s for sit-to-walk, indicated by the purple dots. These overestimates were due to the IMU system missing the first step (verified by observing the video from the RGB camera) and instead detecting the second step, which happened in 19 of the 173 processed trials (ie, in 11% of cases) across 11 participants. Additionally, there was a single large outlier affecting sit-to-walk in phases 1 and 2 caused by misidentification of the peak upward acceleration event.

Figure 7 demonstrates the mechanism by which these failed event detections (ie, failure to detect the first step) took place. In these cases, the IMU did not register a strong upward acceleration signal magnitude, which is widely used to detect initial contact events during walking. The failed detections occurred in trials where the participant completed the movement swiftly, such that their first footstep took place while they were still rising, and the second footstep was more prominent. Where peak upward acceleration was misidentified, there were 2 large upward accelerations registered during the extension phase of the trial, for which the IMU picked out the first peak and the C7 the second, though these peaks were similar in magnitude. This occurred when the participant hesitated or swayed during the rising part of the movement.

A sensitivity analysis was conducted by excluding the 19 trials with failed first step detection. Excluding these trials greatly improved the agreement for outcome metrics reported for overall duration and phase 3 duration. The RMSE for total duration decreased from 0.18 to 0.09 s, and the bias decreased from 0.06 s down to 0.01 s. Meanwhile, the width of the 95% limits of agreement decreased from 0.67 to 0.31 s, and the proportional bias (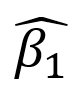) dropped from 0.13 to 0.01. For phase 3 duration, the RMSE dropped from 0.17 to 0.08 s, and the 95% limits of agreement narrowed from 0.66 to 0.28 s, and the bias flipped from 0.02 to –0.03 s. The other outcome metrics remained essentially unchanged, which reflected the fact that they did not depend on precise identification of the movement ending.

**Figure 7:**
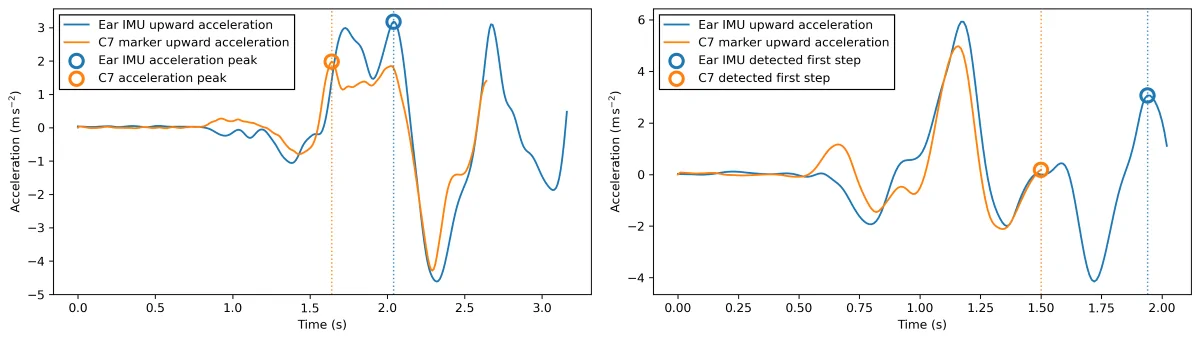
Failure cases observed when identifying phases of the sit-to-walk movement. The left image shows misidentification of peak acceleration, the key transition event between phases 1 and 2, while the right image shows the misidentification of the first step by the IMU, where instead the second step has been picked up. C7: seventh cervical vertebra; IMU: inertial measurement unit.

### Power, Acceleration, and Velocity

The outcome metrics related to power, acceleration, and velocity calculated using the C7 data and the earables data are shown in Table 4.

The fitted slopes for C7 against ear-worn IMUs were of 0.70 for sit-to-stand and 0.68 for sit-to-walk, indicating that a given change in calculated C7 power corresponded to a proportionally larger change in calculated ear-worn IMU power. This is illustrated by the blue and orange best-fit lines in Figure 8.

Figure 9 shows equivalent plots for the peak upward acceleration and velocity recorded by the ear-worn IMUs plotted against those recorded from the C7 marker. These reflect the strong linear relationship between the 2 measurements. However, the values reported from the ear-worn IMUs were systematically higher with significant proportional bias, meaning the optical reference measure could not be directly replicated by the ear-worn system without correction. The peak upward accelerations and velocities tended to be slightly higher for sit-to-walk vs sit-to-stand, with the highest peak upward acceleration recorded as 11.17 vs 8.71 ms^–2^ and peak upward velocity recorded as 1.53 vs 1.31 ms^–1^. While the linear scale factor was similar for sit-to-stand and sit-to-walk for peak upward acceleration (0.73 and 0.74, respectively), it was slightly higher for sit-to-stand for peak upward velocity (0.73 vs 0.65).

**Table 4:** Summary of outcome metrics calculated from the power, upward acceleration, and upward velocity data for sit-to-stand and sit-to-walk.

Activity and outcome		Earable, mean (SD)	C7^a^, mean (SD)	Bias (95% CI)	Lower 95% LoA^b^ (95% CI)	Upper 95% LoA (95% CI)	RMSE^c^	Proportional bias			SDD^d^
								β (95% CI)	Holm *P* value		
Sit-to-stand											
	Peak power (W)	643 (213)	471 (162)	172 (153 to 190)	16 (–5 to 40)	328 (285 to 362)	189	0.29 (0.24 to 0.35)	<.001	91.81	
	Mean power (W)	237 (75)	187 (75)	50 (38 to 63)	–61 (–81 to –39)	162 (133 to 191)	75	–0.04 (–0.20 to 0.11)	.99	103.75	
	Peak upward acceleration (ms^–2^)	3.85 (1.20)	2.80 (0.95)	1.05 (0.93 to 1.18)	–0.04 (–0.22 to 0.15)	2.13 (1.83 to 2.45)	1.18	0.20 (0.13 to 0.28)	<.001	0.83	
	Mean upward velocity (ms^–1^)	0.27 (0.05)	0.18 (0.03)	0.08 (0.07 to 0.09)	–0.02 (–0.05 to 0.00)	0.19 (0.16 to 0.21)	0.10	0.77 (0.62 to 0.93)	<.001	0.07	
	Peak upward velocity (ms^–1^)	0.86 (0.18)	0.65 (0.15)	0.21 (0.19 to 0.22)	0.05 (0.02 to 0.09)	0.36 (0.33 to 0.39)	0.22	0.21 (0.14 to 0.28)	<.001	0.10	
Sit-to-walk											
	Peak power (W)	777 (278)	586 (218)	191 (168 to 215)	–27 (–62 to 7)	410 (361 to 453)	221	0.28 (0.21 to 0.35)	<.001	208.93	
	Mean power (W)	301 (99)	275 (96)	27 (16 to 39)	–87 (–104 to –68)	140 (113 to 168)	64	0.00 (–0.11 to 0.12)	.98	118.31	
	Peak upward acceleration (ms^–2^)	5.25 (1.85)	3.96 (1.44)	1.30 (1.14 to 1.45)	–0.19 (–0.43 to 0.07)	2.78 (2.37 to 3.18)	1.49	0.21 (0.15 to 0.28)	<.001	1.40	
	Mean upward velocity (ms^–1^)	0.22 (0.06)	0.18 (0.05)	0.04 (0.03 to 0.06)	–0.09 (–0.12 to –0.06)	0.17 (0.14 to 0.21)	0.08	0.95 (0.79 to 1.11)	<.001	0.12	
	Peak upward velocity (ms^–1^)	0.96 (0.21)	0.77 (0.18)	0.20 (0.17 to 0.22)	–0.01 (–0.05 to 0.04)	0.40 (0.35 to 0.45)	0.22	0.22 (0.13 to 0.31)	<.001	0.21	

^a^C7: seventh cervical vertebra.

^b^LoA: limits of agreement.

^c^RMSE: root mean squared error.

^d^SDD: smallest detectable difference.

**Figure 8:**
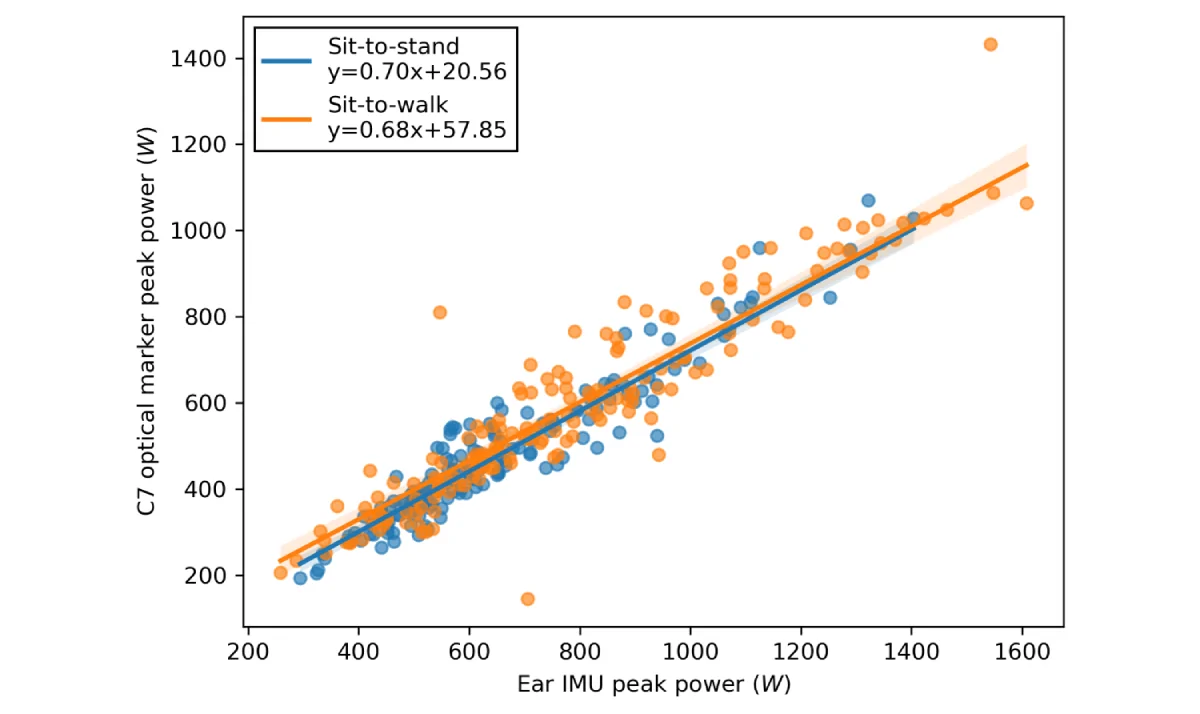
Peak power recorded by the optical marker (y-axis) compared to the ear-worn IMUs (x-axis). C7: seventh cervical vertebra; IMU: inertial measurement unit.

**Figure 9:**
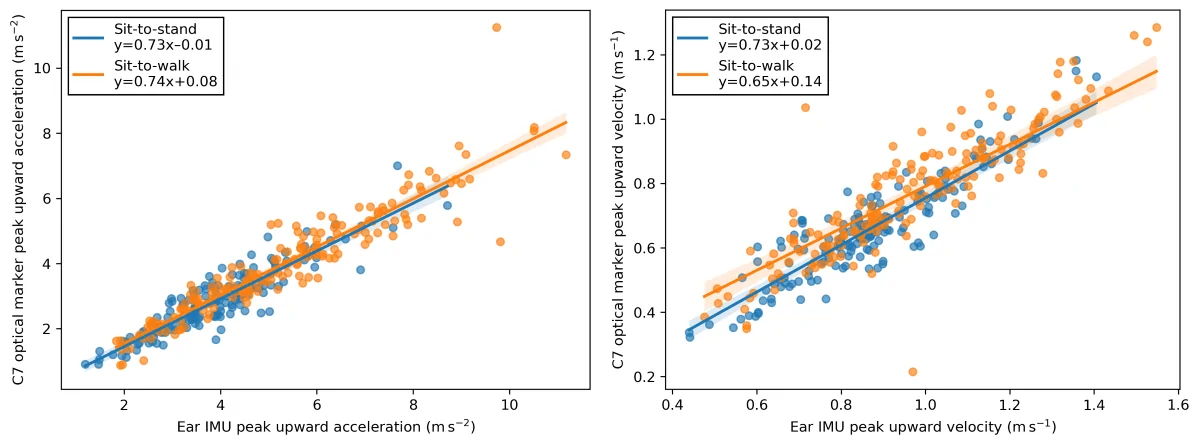
Peak upward accelerations (left) and velocities (right) recorded by the optical marker (y-axis) compared to the ear-worn IMUs (x-axis). C7: seventh cervical vertebra; IMU: inertial measurement unit.

### Exploratory Analysis of Fluency Metrics

The outcome metrics from the exploratory analysis of fluency are shown in Table 5.

**Table 5:** Outcome metrics calculated for the movement fluency measures.

Activity and outcome		Earable, mean (SD)	C7^a^, mean (SD)	Bias (95% CI)	Lower 95% LoA^b^ (95% CI)	Upper 95% LoA (95% CI)	RMSE^c^	Proportional bias		SDD^d^
								β (95% CI)	Holm *P* value	
Sit-to-stand										
	Hesitation	0.12 (0.03)	0.10 (0.03)	0.02 (0.02 to 0.03)	–0.03 (–0.04 to –0.02)	0.07 (0.06 to 0.09)	0.04	–0.28 (–0.41 to –0.15)	<.001	0.07
	Smoothness	14.61 (3.62)	24.10 (7.30)	–9.50 (–10.83 to –8.18)	–21.33 (–24.23 to –18.48)	2.33 (0.40 to 3.98)	11.25	–0.64 (–0.77 to –0.50)	<.001	10.39
Sit-to-walk										
	LDLJ-A^e^	–4.84 (0.23)	–5.21 (0.34)	0.37 (0.31 to 0.45)	–0.30 (–0.56 to –0.05)	1.05 (0.69 to 1.44)	0.50	–0.73 (–0.86 to –0.59)	<.001	0.69
	Hesitation	0.12 (0.04)	0.10 (0.03)	0.02 (0.02 to 0.03)	–0.03 (–0.04 to –0.02)	0.07 (0.05 to 0.10)	0.03	0.10 (0.00 to 0.19)	.13	0.06
	Smoothness	13.81 (4.11)	20.50 (7.16)	–6.65 (–7.72 to –5.62)	–16.79 (–19.03 to –14.35)	3.50 (2.19 to 4.66)	8.42	–0.55 (–0.66 to –0.44)	<.001	10.32
	LDLJ-A	–5.00 (0.27)	–5.99 (0.40)	0.99 (0.91 to 1.07)	0.14 (0.02 to 0.27)	1.84 (1.64 to 2.05)	1.08	–0.86 (–1.02 to –0.70)	<.001	1.02
	Forward velocity reduction	15.40 (36.71)	31.53 (22.99)	–16.45 (–23.52 to –10.71)	–106.35 (–144.30 to –64.74)	73.45 (40.80 to 100.97)	47.72	1.55 (1.42 to 1.68)	<.001	127.14

^a^C7: seventh cervical vertebra.

^b^LoA: limits of agreement.

^c^RMSE: root mean squared error.

^d^SDD: smallest detectable difference.

^e^LDLJ-A: log dimensionless jerk–acceleration.

### Hesitation

The hesitation scores calculated using the method described by Chandler et al [17] showed a relatively small mean bias between the ear-worn IMUs and the optical reference for both sit-to-stand and sit-to-walk (mean bias 0.02, SD 0.03, and RMSE=0.04 and RMSE=0.03, respectively). However, observing the Bland-Altman plots in Figure 10, it is apparent that the largest differences occurred at low values, particularly for sit-to-stand, whereas the difference remained within the limits of agreement even for the large outlier values. This suggests there was a ceiling effect for low values of hesitation.

Conversely, the percentage drop in forward velocity was greatly underestimated by the earables, reporting a mean bias of –16.45 (SD 45.9) and large limits of agreement (–106.35 to 73.45). This is illustrated in Figure 11.

**Figure 10:**
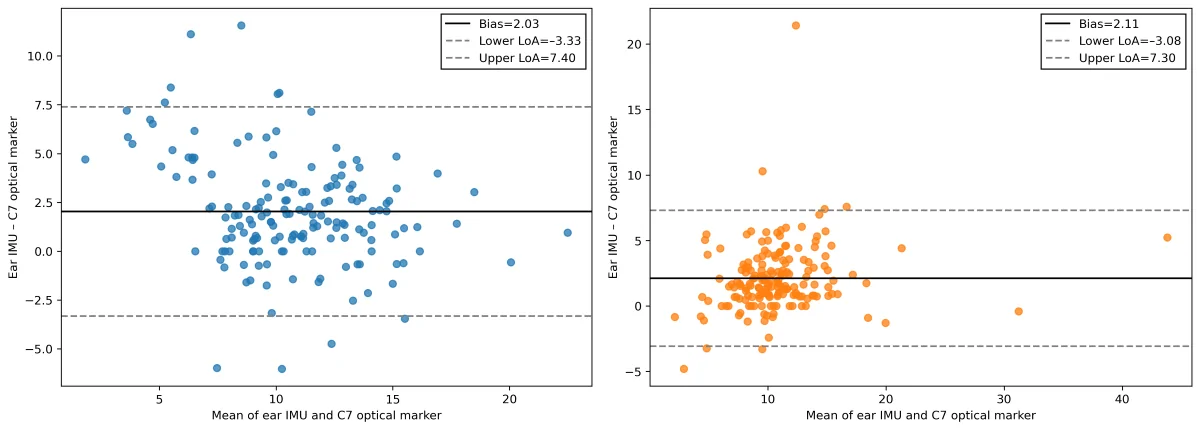
Comparison of hesitation values calculated using data from the earables and the C7 marker for sit-to-stand and sit-to-walk. Hesitation was defined as the difference in time between peak forward velocity and peak upward acceleration for both data sources. C7: seventh cervical vertebra; IMU: inertial measurement unit; LoA: limits of agreement.

**Figure 11:**
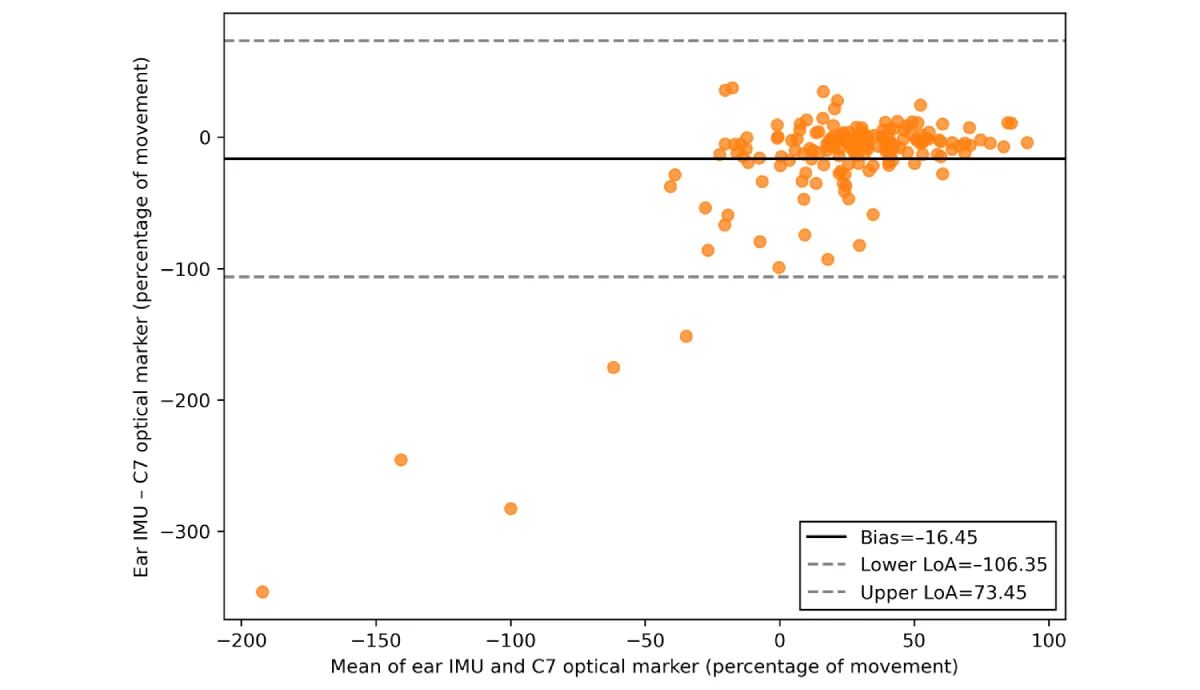
Hesitation for the sit-to-walk when defined as percentage drop in forward velocity. C7: seventh cervical vertebra; IMU: inertial measurement unit.

### Smoothness

The smoothness scores shown in Table 5 indicate the relatively poorer agreement between the LDLJ-A calculated using the earables and the C7 marker (biases of 0.37 and 0.99 and RMSE of 0.50 and 1.08 for sit-to-stand and sit-to-walk, respectively) when compared to simple inflection counting.

### Sensitivity Testing

Table 6 shows the results of the time-series–based anatomical-location analysis performed using the data from the ear-worn IMUs, C7 optical marker, and markers placed on top of the ear-worn IMUs. For all comparisons, the measurement and reference data agreed closely, reporting correlation coefficients ranging from 0.91 to 0.99 and small RMSE values.

For several outcomes, particularly sit-to-walk upward acceleration and upward velocity, agreement was closer between the ear-worn IMU and ear-level optical markers than between the ear-worn IMU and C7 optical marker. This is demonstrated by the higher correlation scores for forward and upward acceleration during sit-to-walk (*r*=0.98 vs *r*=0.94, bias=–0.05 ms^–2^ vs bias=–0.08 ms^–2^; and *r*=0.99 vs *r*=0.92, bias=0.03 ms^–2^ vs bias=0.11 ms^–2^, respectively) and for upward velocity (*r*=0.99 vs *r*=0.91, bias=0.02 ms^–1^ vs bias=0.01 ms^–1^). However, forward velocity showed relatively stronger agreement between the ear- and C7-optical marker signals compared to the ear-IMU and ear-level optical markers (*r*=0.99 vs *r*=0.95, RMSE=0.06 ms^–1^ vs RMSE=0.15 ms^–1^).

**Table 6:** Time-series agreement between ear-worn IMUs^a^, C7^b^ optical reference, and markers placed on top of the IMUs.

Signal and comparison		Mean *r* (95% CI)			RMSE^c^			Mean bias (95% CI)	
		STS^d^	STW^e^	STS		STW	STS		STW
Forward acceleration									
	Ear IMU vs C7	0.96 (0.95-0.97)	0.95 (0.94-0.95)	0.35		0.56	0.00 (–0.02 to 0.01)		–0.10 (–0.13 to –0.07)
	Ear IMU vs optical ear	0.97 (0.91-0.99)	0.98 (0.98-0.99)	0.51		0.29	0.00 (–0.01 to 0.02)		–0.05 (–0.08 to –0.02)
	Optical ear vs C7	0.94 (0.89-0.97)	0.94 (0.92-0.96)	0.68		0.56	0.00 (–0.01 to 0.00)		–0.04 (–0.06 to –0.02)
Upward acceleration									
	Ear IMU vs C7	0.94 (0.93-0.95)	0.94 (0.92-0.95)	0.44		0.74	0.05 (0.04 to 0.06)		0.10 (0.08 to 0.12)
	Ear IMU vs optical ear	0.96 (0.91-0.99)	0.99 (0.99-0.99)	0.75		0.31	0.03 (0.01 to 0.04)		0.03 (0.01 to 0.04)
	Optical ear vs C7	0.91 (0.86-0.95)	0.93 (0.91-0.95)	1.04		0.81	0.00 (–0.00 to 0.00)		0.08 (0.05 to 0.11)
Forward velocity									
	Ear IMU vs C7	0.97 (0.96-0.98)	0.92 (0.90-0.93)	0.1		0.18	–0.04 (–0.07 to –0.02)		–0.06 (–0.08 to –0.04)
	Ear IMU vs optical ear	0.98 (0.96-0.99)	0.95 (0.93-0.97)	0.07		0.15	–0.02 (–0.06 to 0.01)		–0.04 (–0.08 to –0.01)
	Optical ear vs C7	0.98 (0.95-0.99)	0.99 (0.98-0.99)	0.06		0.06	–0.01 (–0.01 to –0.00)		0.01 (0.01 to 0.02)
Upward velocity									
	Ear IMU vs C7	0.92 (0.90-0.93)	0.93 (0.92-0.94)	0.13		0.14	0.08 (0.06 to 0.09)		0.03 (0.02 to 0.05)
	Ear IMU vs optical ear	0.97 (0.93-0.99)	0.99 (0.99-1.00)	0.08		0.05	0.04 (0.02 to 0.07)		0.02 (0.01 to 0.04)
	Optical ear vs C7	0.91 (0.87-0.93)	0.91 (0.88-0.94)	0.11		0.15	0.00 (0.00 to 0.01)		–0.02 (–0.03 to –0.01)

^a^IMU: inertial measurement unit.

^b^C7: seventh cervical vertebra.

^c^RMSE: root mean squared error.

^d^STS: sit-to-stand.

^e^STW: sit-to-walk.

## Discussion

### Principal Findings

This study aimed to evaluate whether ear-worn IMUs can accurately quantify temporal and kinematic metrics of sit-to-stand and sit-to-walk movements when compared to a reference optical motion capture system. Across the investigated metrics, the ear-worn system generally agreed closely with the optical reference; however, this was not universal, and further research is required, particularly for the movement fluency metrics.

The overall and phase durations calculated using the ear-worn IMUs generally agreed with those calculated using the optical reference, exhibiting small systematic biases and especially close agreement for phases 1 and 2 of both movements. While sit-to-stand showed no evidence of proportional bias, this was not the case for sit-to-walk, largely due to a small but notable number of trials missing the first step (indicating the end of the movement). In contrast, the acceleration, velocity, and power metrics were systematically greater when measured from the ear-worn IMUs vs the optical reference, especially when considering peak measurements. The conducted sensitivity analysis suggested that for these measures, much of the disparity arose from the different anatomical measurement location as opposed to sensor-derived measurement error, and the estimated proportionality constants suggested that a given increase at the optical reference corresponded to a slightly larger though consistent increase when measured at ear level.

The exploratory analyses of movement fluency demonstrated more mixed agreement across metrics. Hesitation demonstrated small average bias for the ear-worn IMUs vs the optical reference, though some proportional bias was measured for both movements. Conversely, the alternative forward velocity reduction definition of hesitation found notably poor agreement between the 2 systems, indicating that it could not be reliably implemented by the ear-worn IMUs using the proposed processing method. Lastly, the smoothness metrics suffered from substantial systematic and proportional bias, and require further investigation to determine whether they could be suitably measured using ear-worn IMUs.

### Discussion of Findings

#### Overall Movement and Phase Durations

The finding that the ear-derived overall movement and phase durations agree closely with the optical reference is logical given the nature of these measures. While the other measures rely heavily on the exact composition of the recorded signal—for instance, the maximum recorded acceleration value or the precise count of inflections measured across the whole signal—duration simply requires the system to identify when a particular biomechanical event occurs. The original segmentations proposed by Kerr et al [24] and Schenkman et al [38] are grounded in clear kinematic features and indeed were adapted specifically to suit measurement by an IMU-based system (namely, replacing peak upward force with peak upward acceleration to define the start of phase 2 [extension] and the end of sit-to-walk). By ensuring the phases were divided by events that left prominent landmarks in the IMU signals, these measures were inherently more robust to subtle variations between the signals from the ear-worn IMUs and the optical reference.

A notable exception was the inability of the IMU-based system to reliably detect foot-off events from the recorded data. This unfortunately prevented the system from identifying the durations of phases 3 and 4 in the sit-to-walk movement and presents a significant limitation, as this marks the transition point between rising and initiation of gait. While some previous studies have identified this transition using a threshold in the medio-lateral ground reaction force, others have struggled to identify this, and it is therefore not widely used in a clinical setting [24,73]. The present work was similarly unable to consistently identify a corresponding medio-lateral acceleration or roll movement at the ear level.

Otherwise, the primary failure cases arose from missed detection of the first step of the sit-to-walk movement, which affected 11% (19/173) of trials. Although the upward acceleration peak, which defined the first step, was typically prominent across the trials, this was obscured in cases when participants were not fully upright upon taking this step. While this constitutes a weakness in the system, it is expected that this movement pattern would be less common in populations with more significantly impaired movement, as they would usually exhibit a longer and less fluent transition between rising and initiation of gait. A handful of similar discrete failure cases occurred when identifying the peak upward acceleration defining the transition from phases 2 to 3 in the sit-to-stand movement; however, these were far less common—this is reflected in the comparatively low bias in the durations measured for phases 1 and 2.

The sensitivity analysis performed by excluding trials with failed first step detections reduced the mean bias to within a single sample (0.01, SD 0.08 s) with greatly reduced limits of agreement. This further supports the use of ear-worn IMUs to quantify the duration of both movements and their constituent phases. However, future work should reinforce the reliability of key event detection and investigate instrumenting the sit-to-walk movement with strict placement of the feet on a force plate to investigate whether a relationship between the ear-level and force plate–reported medio-lateral signals exists, or develop other more reliable methods based on IMU-specific features.

#### Acceleration, Velocity, and Power

Unlike the duration metrics, the acceleration and velocity measures relied explicitly on the magnitude of the signal reported by the IMU. The peak upward acceleration and velocity values recorded by the ear-worn IMUs were systematically greater than those measured at the optical reference and demonstrated significant positive proportional bias. Therefore, the values reported by the ear-worn IMUs could not directly replace those reported by the optical reference without the application of a scaling factor.

Direct comparison between the ear-worn IMUs and optical reference was made difficult both due to the difference in sensing modality and anatomical placement. The IMUs directly measured acceleration and then integrated this to calculate velocity, whereas the optical reference directly measured displacement and required consecutive differentiations to calculate velocity and acceleration; in addition, the IMUs were placed on the head while the optical reference was placed on the C7, meaning some disagreement inevitably arose from differences in their respective trajectories. The anatomical-location analysis found markedly high agreement between the IMUs and ear-level markers for upward acceleration (r=0.96-0.99; RMSE=0.31-0.75 ms⁻²) and velocity (r=0.97-0.99; RMSE=0.05-0.08 ms⁻¹). This agreement was particularly strong for sit-to-walk, for which the ear-worn IMU and ear-level optical marker showed lower RMSE than the ear-worn IMU and C7 marker for upward acceleration (0.31 vs 0.74 ms⁻²) and upward velocity (0.05 vs 0.14 ms⁻¹). This suggests that much of the disagreement between the ear-worn IMUs and C7 optical reference could be attributed to measurement location rather than modality.

Comparison with existing studies provides further evidence that anatomical location influences the magnitude of measured acceleration. The peak upward accelerations for sit-to-stand were similar to those recorded at the ear level by Cristiano et al [67] (approximately 4 ms^–2^ from their ear-worn IMU) and slightly lower than Hellec et al [74] (4.65 ms^–2^ from their smart glasses system), though this lies within one SD. Other studies have observed peak upward accelerations of 3.8 (IQR 2.94-4.73) ms^–2^ from a chest-worn IMU [5], 2*.*95±1*.*04 ms^–2^ from an IMU worn on the L3 vertebra [12], and approximately 2.2 ms^–2^ using a CoM calculated using an optical system [27], which reinforce our finding that placing the IMU at the ear overestimates the acceleration of the CoM. Extrapolation of the linear relationship found between the values recorded in the present study at the head and the C7 marker to those placed lower on the trunk would result in a comparable estimate; however, further work should seek to quantify this, particularly as the coefficient was different for sit-to-stand and sit-to-walk, which may be due to intraparticipant differences in movement strategy for these 2 activities.

Similarly, the peak velocities reported by the ear-worn IMUs demonstrated a strong linear relationship with those measured by the C7 marker and were comparable to existing work that reported peak upward velocities between 0.6 and 0.8 ms^–1^ [12,27,57]. However, the mean velocities were lower than those reported by other work [5,47], potentially due to the relatively low seat height used in this study, which has been shown to result in lower mean vertical velocities during sit-to-stand [30].

The greater peak upward accelerations and velocities for sit-to-walk compared to sit-to-stand were potentially caused by the increased need to generate momentum to initiate walking, as suggested by Kerr et al [24], though this may have been purely a result of the greater emphasis placed on the fast completion of the exercise. To the best of the authors’ knowledge, there are no normative values available to compare with for the sit-to-walk movement, as prior work has focused more on phase timings and relative horizontal velocities.

As the power measures are derived directly from combining acceleration and velocity measures, it follows that a similar relationship was found. The relative sparsity and wide variety of definitions of power metrics make it difficult to compare the values obtained in this study with existing work, especially as this has focused on older participant cohorts. Zhang et al [13] recorded similar peak powers of 763*.*1 (SD 315.2) W from an accelerometer worn in a pendant form factor during in-clinic sit-to-stand tests. While this represents the closest form factor to the earables out of existing works, the authors used the normalized 3-axis acceleration in their power calculation, which would result in an overestimate compared to our method. Regterschot et al [57] also recorded comparable power values of 645*.*3 (SD 187.3) W during “fast” sit-to-stand movements with an IMU placed at the hip. However, Atrsaei et al [5] recorded substantially lower peak power values of 189.7 W from a chest-worn IMU for a similar cohort of 458 nonfalling older adults during a Five-Times sit-to-stand test, which would appear to contradict ours and other work. Given that the velocities recorded in their work were in fact greater than in the present work (1.12, IQR 0.95-1.27 ms^–1^) while reporting similar accelerations (3.8, IQR 2.94-4.73 ms^–2^), this is likely due to a difference in the power calculation performed.

Overall, it seems that the acceleration, velocity, and power metrics measured by the ear-worn system would be feasible, though they require vastly more data captured at the ear level from typically functioning and movement-impaired populations to determine suitable clinically useful thresholds.

#### Movement Fluency

The movement fluency metrics were calculated to explore whether measures of movement quality as opposed to temporal and kinematic measures alone could be calculated from the ear-worn IMUs. The 4 measures calculated in this work capture different, though complementary, aspects of movement quality, meaning in turn that their validity varied when compared with the optical reference.

Hesitation captures the temporal separation between biomechanically significant forward and upward movement events, providing a means to objectively describe the transition of forward momentum into vertical displacement. Given that this calculation relies on identifying prominent extrema in the acceleration and velocity signals, which is analogous to the phase identification discussed earlier, it followed that the agreement between the ear-worn IMUs and optical reference was comparatively closer for hesitation than the other fluency measures. Mean bias was only 0.02 (SD 0.03) for both sit-to-stand and sit-to-walk movements, with RMSEs of 0.04 and 0.03. Significant proportional bias was observed for sit-to-stand (β=–0.28, 95% CI –0.41 to –0.15; *P*<.001) but not sit-to-walk (β=0.10, 95% CI 0.00 to 0.19; *P*=.130). The sit-to-stand relationship appeared to reflect greater disagreement at lower hesitation values, potentially indicating a ceiling effect that affected lower movement durations (and therefore faster transitions). The existing work that has included hesitation has done so with more highly impaired populations—in the case of Chandler et al [17], these were those who had endured a stroke within the last 3-42 days, and for Edwards et al [75], prosthetic patients. In these cases, the participants took longer to perform the movements and did so with more inherent variability, which allowed meaningful conclusions to be drawn from the hesitation metric. Future work should therefore seek to apply the hesitation calculation method used with the ear-worn IMUs to these populations to confirm that the system can effectively capture this difference in their movement.

The alternative hesitation measure—percentage drop in forward velocity during the sit-to-walk movement—demonstrated markedly lower agreement with the optical reference, encapsulated by a mean bias of –16.45 (SD 45.9) percentage points (95% CI –23.52 to –10.71), RMSE of 47.72 percentage points, and wide 95% limits of agreement from –106.35 to 73.45 percentage points. The anatomical-location sensitivity analysis found very strong agreement between the ear-level optical markers and C7 marker for forward velocity (mean r=0.99, 95% CI 0.98 to 0.99; RMSE=0.06 m/s; mean bias=0.01 m/s, 95% CI 0.01 to 0.02), whereas agreement between the ear-worn IMU and ear-level optical marker was lower (mean r=0.95, 95% CI 0.93 to 0.97; RMSE=0.15 m/s; mean bias=–0.04 m/s, 95% CI –0.08 to –0.01). Contrary to the upward acceleration and velocity measures, this suggested that the disagreement arose primarily due to IMU measurement error rather than anatomical separation between the ear and C7 locations. This is likely explained by errors introduced during integration of the forward acceleration signal, which could not be mitigated via linear drift correction as the movement did not end in a stationary pose.

The relatively low RMSE between the earables and the C7 marker for the number of inflections in the jerk signals ostensibly contradicted the relatively high RMSE for LDLJ-A. This could have been due to a ceiling effect, as the number of peaks recorded was generally much lower and less varied than in the work by Chandler et al [17], suggesting generally smoother movement. This would be expected given the higher functional ability of the participant group in our study. However, given that the calculation of jerk requires 3 successive differentiations of the marker trajectory vs only a single differentiation of the IMU signal, it is questionable whether the marker data act as a suitable reference measurement. This is especially true given the high prevalence of artifacts in optical marker data and the susceptibility of LDLJ metrics to noise [71,76]. Indeed, Brasiliano et al [77] recently found similarly poor correlation scores when comparing LDLJ metrics between a trunk-worn IMU and an optical motion capture system during gait.

The results of this study remain inconclusive regarding the accuracy of measured movement smoothness during sit-to-stand and sit-to-walk with earables. Smoothness provides a quantitative measure of movement fluency, which is not reflected in other metrics but is readily interpretable to a human observer; however, the absolute scores have been shown to be heavily task-specific [17,32,71]. To this end, future work should seek to quantify movement smoothness during sit-to-stand and sit-to-walk using an IMU-based system while performing the transitions with different levels of fluidity to both confirm the discriminatory power of the smoothness metric and to provide normative values that can be compared in future studies.

### Implications for Clinical Assessment

While the present work is focused on validating metrics derived from ear-worn IMUs against an optical reference, this alone does not imply clinical utility. Sit-to-stand and sit-to-walk movements are included in multiple clinical tests with a range of outcome metrics: the Five-Times-Sit-to-Stand and the TUG rely on total duration, while the Amputee Mobility Predictor, Tinetti Balance Scale, and Freezing of Gait Questionnaire explicitly require the assessment of hesitation during sit-to-stand [20,21,78-80]. However, these tests typically provide subjective guidance for clinicians as opposed to quantitative thresholds, meaning there is no widespread consensus regarding acceptable clinical thresholds for determining functional impairment or falls risk.

The outcome metrics reported in this study included smallest detectable difference (SDD), which represents the minimum magnitude of change that must be exceeded to confidently distinguish it from expected intraparticipant measure variability. For overall movement duration, the SDDs were 0.28 s for sit-to-stand and 0.29 s for sit-to-walk movements, suggesting that any clinically meaningful differences would need to exceed a few tenths of a second to be detectable by the ear-worn system. To provide context, existing work has estimated clinically meaningful changes in Five-Times-Sit-to-Stand to be between 0.72 and 3.0 s in a heterogeneous cohort of patients who had stroke [81]. While not directly comparable with a single sit-to-stand movement, this suggests that clinically relevant changes may exceed the measurement variability found in the present study. In contrast, the smoothness and percentage reduction in forward velocity measures had large SDDs of 10.32 and 134.84 for sit-to-walk, suggesting that even if a minimum clinically meaningful difference had already been determined for these movements, the present implementation would potentially not be sufficiently sensitive to detect this over measurement noise without refinement.

Achieving satisfactory measurement accuracy and repeatability using ear-worn IMUs could provide potential for longitudinal quantitative movement assessment in neurological or otherwise movement-impaired populations. Existing work has sought to use gait and movement features derived from wearable sensors as digital biomarkers, notably for the detection of Parkinson disease, which is characterized partly by its distinctive impact on the patient’s movement quality [82]. The widespread use of ear-worn devices, many already containing IMUs, means that the measures described in the present work could be readily adopted into existing form factors that individuals may already be wearing. This would allow the broader collection of data required to establish normative and condition-specific clinical thresholds for the more feasible measures discussed in the present work, which could be reliably replicated between ear-worn devices.

### Limitations

This study has several limitations. A key limitation is the difference in anatomical location between the ear-worn IMUs and the C7-placed optical marker, which made it difficult to isolate whether disagreement was caused by sensor measurement error as opposed to anatomical-location differences. While the sensitivity analyses attempted to address this, they were only conducted on a small subset of participants, meaning that these were indicative rather than a definitive validation.

Another key limitation relates to the algorithm design for calculating the outcome metrics. Several important features—notably movement start or end, phase transitions, and filter parameters—were calculated using empirically defined thresholds and search windows. Although the participant group contained some individuals with underlying movement conditions, there was insufficient variation to determine whether the same settings would transfer to those with substantially differing movement patterns. Indeed, a select few failure cases were isolated and discussed in the Results section, suggesting that some additional robustness measures should be added and tested before clinical application. Similarly, an inherent drawback of using IMUs to measure velocity-based metrics is the need to integrate acceleration signals, which introduces a high susceptibility to drift. While assuming stationary start and end poses for sit-to-stand largely mitigated these issues, this may not be valid when analyzing daily living scenarios as opposed to controlled clinical tests, and indeed this was not possible at all for the sit-to-walk movements.

A final major limitation to note is the relative lack of clinically meaningful thresholds for some of the outcome metrics—most evidently for power and the movement fluency metrics. This is further compounded by the lack of consensus on the definition of each movement phase and methodology for calculating power. Without this information, it was difficult to draw conclusions about the clinical acceptability of any observed measurement errors between the ear-worn IMUs and the optical reference. Future work should focus on collecting measurement data from normative and specific movement-impaired populations to establish suitable clinical thresholds, which in turn will enable the adoption of ear-level and other IMU-based analysis approaches into clinical practice.

### Conclusions

This study demonstrates the feasibility of deriving a broad range of sit-to-stand and sit-to-walk measures from ear-worn IMUs. Movement and phase durations generally demonstrated small systematic differences compared with the optical reference, though occasional misidentification of key temporal features produced substantial trial-level errors. Acceleration, velocity, and power outcomes demonstrated magnitude-dependent disagreement, though it was possible to quantify a proportionality constant to map a given change in the reference measurement to the change in ear-worn measurement. A small anatomical-location sensitivity analysis demonstrated that some differences between the ear-worn IMUs and C7 reference reflected biomechanical differences in motion between the head and upper trunk rather than sensing modality alone. Of the exploratory fluency measures, hesitation showed relatively small mean differences, whereas smoothness and forward velocity reduction demonstrated greater disagreement and require further methodological development.

These findings provide a methodological basis to derive quantitative summary measures of sit-to-stand and sit-to-walk movements using ear-worn IMUs. The results identify which measures appear sufficiently robust to warrant subsequent clinical investigation and where further improvements in event detection and signal processing are required. Integration of these methods into existing ear-worn form factors could ultimately facilitate unobtrusive, repeated assessment of functional movement outside specialized motion-analysis environments, mirroring the advances made in ear-worn gait analysis. Future work should therefore evaluate the reliability, responsiveness, and clinical validity of the most promising outcomes in older and movement-impaired populations, including the establishment of normative and clinically meaningful thresholds, with the long-term aim of evaluating their utility for assessing functional impairment and fall risk.

## Abbreviations

CoM: center of mass
C7: seventh cervical vertebra
IMU: inertial measurement unit
LDLJ: log dimensionless jerk
LDLJ-A: log dimensionless jerk–acceleration
RGB: red, green, and blue
RMSE: root mean squared error
SDD: smallest detectable difference
TUG: timed up-and-go

## Data Availability

The datasets presented in this paper are not readily available because the data are part of an ongoing study. Requests to access the datasets should be directed to TF.
